# Linderone Isolated from *Lindera erythrocarpa* Exerts Antioxidant and Anti-Neuroinflammatory Effects via NF-κB and Nrf2 Pathways in BV2 and HT22 Cells

**DOI:** 10.3390/ijms24087569

**Published:** 2023-04-20

**Authors:** Zhiming Liu, Chi-Su Yoon, Hwan Lee, Hyeong-Kyu Lee, Dong-Sung Lee

**Affiliations:** 1 College of Pharmacy, Chosun University, Dong-gu, Gwangju 61452, Republic of Korea; lzmqust@126.com (Z.L.);; 2 Natural Medicine Research Center, Korea Research Institute of Bioscience & Biotechnology (KRIBB), Cheongju-si 28116, Republic of Korea; 3 Department of Chemistry, University of Florida, Gainesville, FL 32611, USA

**Keywords:** linderone, HT22 hippocampal cells, mouse microglia BV2, protection, anti-neuroinflammation

## Abstract

Linderone is a major compound in *Lindera erythrocarpa* and exhibits anti-inflammatory effects in BV2 cells. This study investigated the neuroprotective effects and mechanisms of linderone action in BV2 and HT22 cells. Linderone suppressed lipopolysaccharide (LPS)-induced inducible nitric oxide synthase, cyclooxygenase-2, and pro-inflammatory cytokines (e.g., tumor necrosis factor alpha, interleukin-6, and prostaglandin E-2) in BV2 cells. Linderone treatment also inhibited the LPS-induced activation of p65 nuclear factor-kappa B, protecting against oxidative stress in glutamate-stimulated HT22 cells. Furthermore, linderone activated the translocation of nuclear factor E2-related factor 2 and induces the expression of heme oxygenase-1. These findings provided a mechanistic explanation of the antioxidant and anti-neuroinflammatory effects of linderone. In conclusion, our study demonstrated the therapeutic potential of linderone in neuronal diseases.

## 1. Introduction

Neurodegenerative diseases, such as Parkinson’s disease and Alzheimer’s disease, are often associated with neuroinflammation. As an important immune response, neuroinflammation protects the body from damage by eliminating harmful factors and repairing cells and tissues. However, excessive neuroinflammation can lead to severe neurological dysfunction [1,2,3]. Microglia are key immune cells that mediate inflammation in the central nervous system [4,5,6]. Under the stimulation of lipopolysaccharide (LPS), microglia produce prostaglandin E2 (PGE_2_) and nitric oxide (NO) through the expression of proinflammatory proteins, inducible nitric oxide synthase (iNOS), and cyclooxygenase (COX-2), resulting in inflammatory-related neurological dysfunction [7,8,9]. In addition, nuclear factor κB (NF-κB) signal transduction is crucial in the process of microglia-mediated neuroinflammation. Nuclear factor-κB (NF-κB)/Rel proteins include NF-κB2 p52/p100, NF-κB1 p50/p105, c-Rel, RelA/p65, and RelB. These proteins act as dimerization transcription factors, among which RelA/p65 is an important type of NF-κB/Rel protein and a key target for regulating inflammatory responses in cells [10]. Under unstimulated conditions, NF-κB is a complex composed of p65 and I-κB subunits, and exists in the cytoplasm [11]. However, LPS induction will lead to the phosphorylation of I-κB, leading to a structural change of the p65/I- κB complex, and the p65 subunit will be dissociated and transferred into the nucleus to affect the transcription of inflammatory factors [12]. After that, the free p65 subunit is transported to the nucleus, where it induces inflammatory factors such as tumor necrosis factor (TNF)-α and interleukin (IL)-6. Therefore, one strategy for inhibiting microglial inflammation is to regulate NF-κB translocation [13,14,15].

Another important pathogenesis of neurodegenerative diseases is oxidative stress [16,17,18]. Oxidative imbalances lead to imbalances in the antioxidant system, leading to the production of reactive oxygen species (ROS) [19]. Rising levels of reactive oxygen species can lead to cell apoptosis, damage proteins and organelles, damage mitochondrial membranes, and lead to neuronal cell death [20]. As a key regulator of the antioxidant response, nuclear factor E2 related factor 2 (Nrf2) can promote the expression of antioxidant enzymes, including heme oxygenase (HO)-1 [21]. HO-1 has strong antioxidant and anti-inflammatory effects [22]. In addition, the activation of the Nrf2/HO-1 pathway can reduce the NF-κB-mediated inflammatory response to prevent neuronal cell death. Therefore, the Nrf2/HO-1 pathway has been a therapeutic target for neuroprotective medicine [23,24].

*Lindera erythrocarpa* (family Lauraceae) is widely distributed in Korea, Japan, and China [25]. Our previous study identified the fungal metabolite linderone in *L. erythrocarpa* and found that it inhibited NO overproduction in lipopolysaccharide (LPS)-treated BV2 cells [26]. However, little research has been conducted on the neuroprotective effects of linderone.

In this study, we therefore investigated the anti-neuroinflammatory effects of linderone in LPS-stimulated BV2 cells. We also examined the protective effects of linderone in glutamate-induced HT22 cells.

## 2. Results

### 2.1. Effects of Linderone on BV2 and HT22 Cell Viability

**Figure 1:**
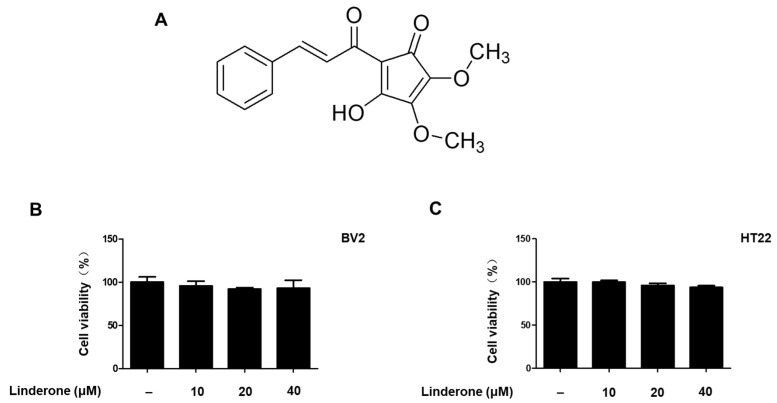
Linderone chemical structure (**A**) and effects on cell viability. BV2 (**B**) and HT22 (**C**) cells were incubated with various concentrations for 24 h. The MTT assay was used to determine cell viability.

Linderone was isolated from the leaves of *L. erythrocarpa Makino* (see Figure 1A for chemical structure). To determine the cytotoxic effects of linderone, we treated HT22 and BV2 cells with various linderone concentrations, followed by a 3-(4,5-dimethylthiazol-2-yl)-2,5-diphenyltetrazolium bromide (MTT) assay. The results showed that 10–40 μM was a safe concentration range (Figure 1B,C). Thus, we set the maximum treatment concentration of linderone at 40 μM for subsequent experiments.

### 2.2. Effects of Linderone on Inflammatory Factors in BV2 Cells

**Figure 2:**
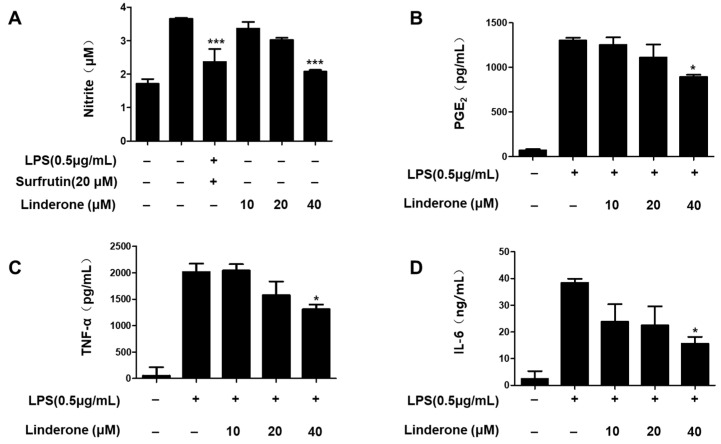
Effects of linderone on nitrite (**A**), PGE_2_ (**B**), TNF-α (**C**), and IL-6 (**D**) levels in LPS-induced BV2 cells. Cells were pretreated with linderone for 2 h and cultured for 24 h with LPS. * *p* < 0.05, *** *p* < 0.001 compared with LPS-treated group. Sulfuretin at a concentration of 20 μM was the positive control.

An LPS-induced BV2 cell was established for measuring nitrite production. We used sulfuretin (20 μM) as a positive control because its anti-inflammatory effect is excellent. The effect of linderone on nitrite levels was significant at 40 μM (Figure 2A). We demonstrated that prostaglandin E2 (PGE_2_) production was inhibited by linderone (Figure 2B). Linderone also inhibited IL-6 and TNF-α production (Figure 2C,D). Thus, linderone significantly reduced the level of inflammatory factors in LPS-induced BV2 cells.

**Figure 3:**
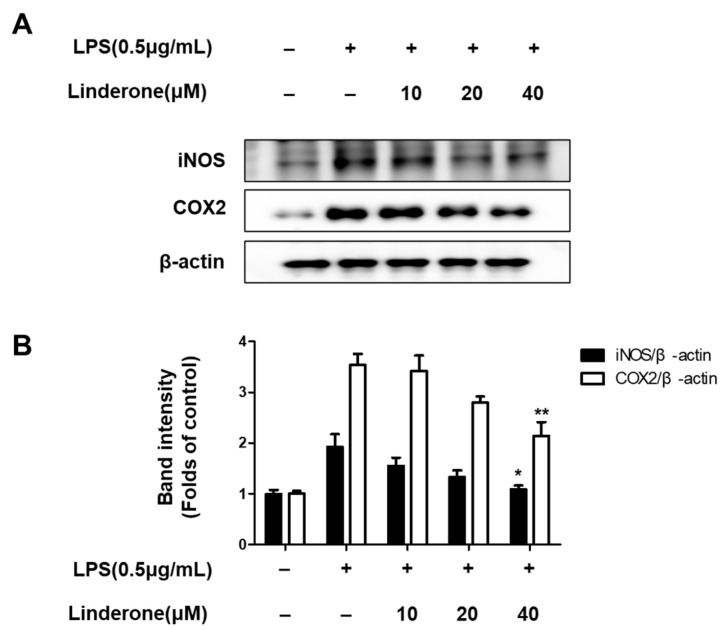
Expression of iNOS and COX-2 (**A**,**B**) in BV2 cells. Cells were pretreated for 2 h with linderone and cultured for 24 h with LPS. Band intensities were normalized to β-actin. * *p* < 0.05, ** *p* < 0.01 compared with LPS-treated group.

Furthermore, western blots of inflammation-related protein expression showed that both iNOS and COX2 expression rose in LPS-treated cells, and linderone pretreatment inhibited this LPS-induced increase (Figure 3).

### 2.3. Effects of Linderone on Regulation of NF-κB (p65) Pathway in BV2 Cells

**Figure 4:**
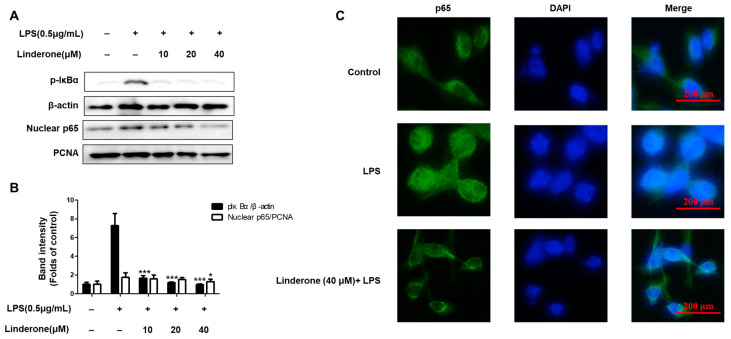
Effects of linderone on NF-κB (**A**,**B**) and p65 localization (**C**) in BV2 cells. Cells were pretreated with linderone for 2 h and cultured with LPS for 1 h. * *p* < 0.05, *** *p* < 0.001 compared with LPS-treated group.

The cytosolic and nuclear fractions from LPS-induced BV2 cells were extracted to explore the inhibitory effect of linderone on the NF-κB pathway. We then measured the protein levels with western blotting. Linderone inhibited p-IκBα expression and p65 nuclear translocation (Figure 4A,B). Using immunofluorescence, we also compared p65 NF-κB expression in LPS-treated cells with and without the linderone treatment (Figure 4C). Linderone reduced the LPS-induced increase in p65 nuclear translocation. These results suggest that linderone blocks the LPS-induced production of neuroinflammatory cytokines through inhibiting the NF-κB pathway.

### 2.4. Effects of Linderone on Glutamate-Induced Oxidative Stress in HT22 Cells

**Figure 5:**
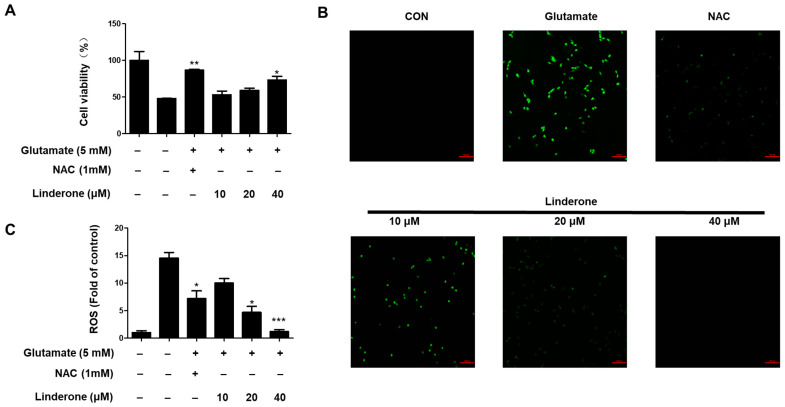
Neuroprotective effect of linderone on HT22 cells. After pretreated with linderone for 2 h, cell viability (**A**) was analyzed in the absence or presence of glutamate for 24 h; ROS generation (**B**) was detected using fluorescence microscope (original magnification: ×100); and fluorescence intensities were quantified using ImageJ software (**C**) * *p* < 0.05, ** *p* < 0.01, *** *p* < 0.001 compared with control group. N-acetyl-cysteine (NAC, 1 mM) was the positive control.

Linderone was treated for proving neuroprotective effects against oxidative stress-induced cytotoxicity in glutamate-treated HT22 cells. Linderone exerted neuroprotective effects in glutamate-treated HT22 cells in a concentration-dependent manner (Figure 5A). Furthermore, the glutamate treatment group produced significantly more ROS than the control group (Figure 5B). Linderone, on the other hand, significantly reduced ROS production (Figure 5C).

### 2.5. Effects of Linderone on Nrf2/HO-1 Pathway in HT22 and BV2 Cells

**Figure 6:**
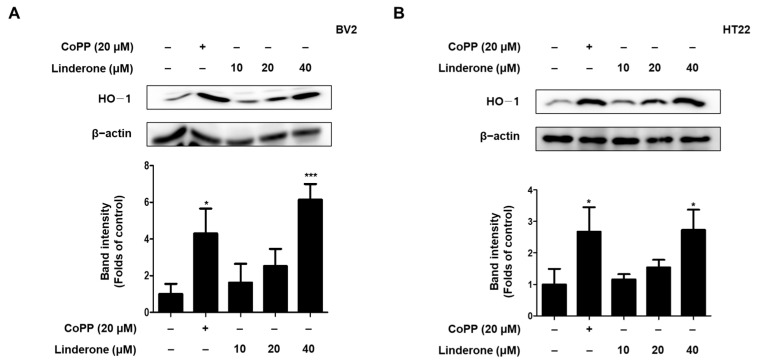
Effects of linderone on HO-1 expression in BV2 (**A**) and HT22 cells (**B**). Cells were treated with linderone or CoPP for 12 h. * *p* < 0.05, *** *p* < 0.001 compared with control group.

To determine whether HO-1 was the source of linderone’s antioxidant effect, we treated BV2 and HT22 cells with various linderone concentrations for 12 h before performing western blotting. Cobalt protoporphyrin (CoPP) served as a positive control. Linderone significantly increased HO-1 expression in BV2 and HT22 cells (Figure 6).

**Figure 7:**
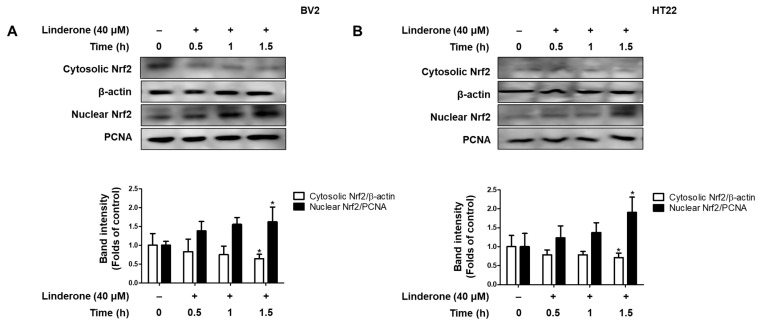
Effects of linderone on Nrf2 in BV2 (**A**) and HT22 cells (**B**). Cells were treated with linderone at different time points. * *p* < 0.05 compared with control group.

We also explore the effect of linderone on Nrf2 activation. We measured Nrf2 expression every 0.5 h and observed that cytosolic Nrf2 transfers to the nucleus in BV2 and HT22 cells after linderone administration (Figure 7).

**Figure 8:**
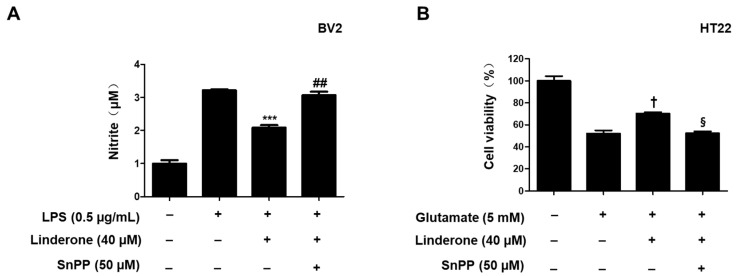
Inhibitory effects of linderone on nitrite production through regulating HO-1 activity in BV2 (**A**) and HT22 cells (**B**). Cells were pretreated with SnPP or linderone and then stimulated for 24 h with LPS or glutamate. *** *p* < 0.001 vs. LPS group. ^##^ *p* < 0.01 vs. LPS and linderone group. ^†^ *p* < 0.05 compared with glutamate group. ^§^ *p* < 0.05 vs. glutamate and linderone group.

Tin protoporphyrin-IX (SnPP), a selective HO-1 inhibitor, was used to further examine whether the neuroprotective effects of linderone were related to the Nrf2/HO-1 pathway. Nitrite concentration was lower in the linderone group compared to those treated with both linderone and SnPP (Figure 8A). The neuroprotective effects were also stronger in the linderone-treated HT22 cells compared to those treated with both linderone and SnPP (Figure 8B). Additionally, SnPP treatment reversed the anti-inflammatory and neuroprotective effects of linderone, suggesting that HO-1 expression regulates linderone’s effects.

## 3. Discussion

Long used as a herbal medicine, *L. erythrocarpa* has been found to exhibit antioxidant, anti-inflammatory, anticancer, and antifungal activities. One of the main compounds in *L. erythrocarpa* is linderone, a cyclopentadione that shows anti-inflammatory effects on BV2 cells. Linderone is a compound belonging to the cyclopentadione class. Reports have shown that linderone not only has the ability to scavenge free radicals such as DPPH or ABTS [27], but can also inhibit NO production in RAW264.7 [28]. However, there are no papers about the regulation of neuronal effects using linderone. Here, we verified that this characteristic of linderone confers neuroprotection.

First, the cell viability experiment determined the safe concentration range of linderone (Figure 1). Next, the effects of linderone on neuroinflammation were confirmed in LPS-induced BV2 cells. Our results show that linderone can significantly inhibit the production of nitrite and PGE_2_ (Figure 2A,B). Nitrite and PGE_2_ are important inflammatory mediators produced by microglia. Nitrite is mediated by iNOS and then destroys the cell membrane of neurons, damaging DNA transcription and protein synthesis [28]. PGE_2_ is a COX-2 product that also plays an important role in neuroinflammation [29]. Other proinflammatory cytokines activated by NF-κB, including TNF- α and IL-6, were also significantly inhibited by linderone (Figure 2C,D). In the meantime, a western blot analysis showed that linderone also inhibited LPS-induced iNOS and COX-2 expression (Figure 3). NF-κB is a key regulatory protein of the aforementioned inflammatory factors, so the effect of linderone on the NF-κB pathway was also determined. As a key protein in the response of microglia to stimulation under ischemic, traumatic, neurotoxic, or inflammatory conditions [30], NF-κB activation contributes to neuronal damage and promotes the development of neurodegenerative diseases [31,32,33]. The results suggest that linderone significantly inhibited the nuclear translocation of p65 (Figure 4). In summary, these results demonstrate that linderone can inhibit inflammation via the NF-κB pathway in LPS-stimulated BV2 cells.

Neuronal apoptosis in neurodegenerative diseases is a direct cause of morbidity, and high concentrations of glutamate can induce neuronal apoptosis through oxidative stress [34]. Therefore, we studied the neuroprotective effect of linderone on glutamate-induced HT22 cells. The results show that linderone significantly improved glutamate-induced damage in HT22 cells and increased cell survival (Figure 5A). After that, our study measured ROS in HT22 cells using a fluorescence microscope. In glutamate-induced neuronal cells, the activation of ionic glutamate receptors leads to excessive ROS formation, ultimately leading to cell death [35]. The results show that Lindelun reversed the ROS produced by HT22 cells induced by glutamate (Figure 5B,C). This result indicates that ninhydrin can protect HT22 cells by inhibiting oxidative stress. The Nrf-2/HO-1 pathway is crucial in neuroprotective strategies [36]. The activation of HO-1 can protect neurons from oxidative damage [37]. Our results indicate that indenone induces HO-1 expression in both BV2 and HT22 cells (Figure 6A,B). On the other hand, Nrf2 is a negative regulator of HO-1, and after activation, it enters the nucleus for transcription and induces the production of HO-1 [38]. We found that linderone treatment decreased the content of Nrf2 in the cytoplasm, while increasing the content of Nrf2 in the nucleus (Figure 7). This result indicates that linderone can activate the Nrf2/HO-1 pathway in BV2 and HT22 cells. We also used SnPP, the inhibitor of HO-1, to verify this point. As expected, linderone reduced the nitrite levels in BV2 cells and protected HT22 cells from death, while SnPP reversed these beneficial effects (Figure 8). Therefore, it can be concluded that linderone significantly targets Nrf2/HO-1 to exert protective effects on HT22 cells. In this study, we only focused on the mechanism of cell protective effects or anti-neuroinflammation by linderone using the two cell lines, BV2 and HT22 cells; however, the neuroprotective effects in vivo should be further studied.

## 4. Materials and Methods

### 4.1. Materials

Cell reagents were purchased from Gibco BRL Co., Ltd. (Grand Island, NY, USA). Primary antibodies were purchased from Cell Signaling Technology (Danvers, MA, USA); ELISA kits were purchased from R&D Systems Inc., (Minneapolis, MN, USA). Secondary antibodies were purchased from Millipore (Billerica MA, USA). Other reagents were purchased from Sigma-Aldrich (St. Louis, MO, USA).

### 4.2. Cell Culture and Cell Viability Assays

BV2 cells were incubated in RPMI1640 containing 1% penicillin-streptomycin and 10% FBS. HT22 cells were cultured in DMEM containing 10% FBS and 1% penicillin-streptomycin, under a humidified atmosphere of 5% CO_2_ and a temperature of 37 °C. Cell viability was measured with the MTT assay.

### 4.3. Measurement of NO Generation

Equal volumes of cell supernatant and Griess reagent were mixed for nitrite level determination.

### 4.4. PGE_2_ Assay

The culture supernatant was collected to determine the PGE_2_ levels per sample using a commercially available kit.

### 4.5. Assays for IL-6 and TNF-α

The supernatant of the BV2-cultured medium was collected to determine IL-6 and TNF-α levels using the ELISA method.

### 4.6. Western Blotting Analysis

Cells were lysed using a RIPA buffer. Protein was mixed with the sample loading buffer, then transferred onto nitrocellulose membranes, which were incubated in skimmed milk in order to block non-specific protein binding. Primary antibodies were treated at 4 °C overnight.

### 4.7. p65 Localization

BV2 cells were seeded on Lab-Tek II chamber slides and then treated with linderone or LPS. Formalin was used for fixing cells. Next, the p65 antibody and Alexa Fluor 488 were treated. DAPI was used to visualize nucleifor. Finally, cells were observed and photographed using a fluorescence microscope (Provis AX70; Olympus Optical Co., Tokyo, Japan).

### 4.8. Determination of ROS Levels in HT22 Cells

HT22 cells were incubated with DCFH-DA (10 μM) for 20 min. After PBS washing, cells were observed and photographed under a fluorescence microscope (Provis AX70; Olympus Optical Co., Tokyo, Japan).

### 4.9. Statistical Analysis

Data are shown as the average and standard deviation of three independent experiments. A one-way ANOVA method was adopted to analyze data using GraphPad Prism version 5.01 (GraphPad Software Inc., San Diego, CA, USA).

## 5. Conclusions

In conclusion, this study suggests linderone can suppress the activation of the NF-κB pathway in BV2 cells. Additionally, linderone activates the Nrf2/HO-1 pathway in both BV2 and HT22 cells. The strong effect of linderone can be attributed to these dual mechanisms. Our findings provide valuable evidence that linderone can be a potential therapeutic target for neurodegenerative diseases.

## Data Availability

Data supporting the findings of this study are available upon request from the corresponding author.
